# Acute Fulminant Cerebral Edema Caused by Influenza Type B in an 18-Year-Old Female: A Rare Case

**DOI:** 10.7759/cureus.45501

**Published:** 2023-09-18

**Authors:** Luis E Santiago, Ali Tariq Alvi, Zahid Nadeem, Ali Chaudhry

**Affiliations:** 1 Internal Medicine, HCA Florida Northwest Hospital, Margate, USA; 2 Internal Medicine, HCA Florida Westside Hospital, Plantation, USA; 3 Critical Care, HCA Florida Northwest Hospital, Margate, USA

**Keywords:** acute respiratory distress syndrome (ards), brain death, critical care, influenza myocarditis, cerebral edema, influenza b, acute fulminant cerebral edema

## Abstract

Most influenza B infections are self-limited, but in some instances, they can cause substantial morbidity and mortality due to complications. Acute fulminant cerebral edema (AFCE) is one of the rare complications. AFCE, a consequence of acute encephalitis, presents as acute onset of alteration in mental status, seizure, and/or headache followed by rapidly progressive encephalopathy, often leading to death. The exact pathophysiology of AFCE is unknown, but many pathomechanisms have been proposed. We present a case of an 18-year-old female in excellent physical condition who presented with respiratory insufficiency after being recently diagnosed with influenza B infection. Three days later, she developed acute encephalopathy, leading to brain death. To our knowledge, this rare case of AFCE developing following influenza B infection is the first reported case outside the pediatric population.

## Introduction

Although most influenza B infections, in pediatric and young healthy adults, are asymptomatic or mild, these infections can occasionally cause substantial morbidity and mortality due to complications. These include pneumonia, cardiac complications, central nervous system complications, myositis, multisystem organ failure, concomitant infection, and acute respiratory distress syndrome (ARDS), among others [1]. Acute fulminant cerebral edema (AFCE) is one of the least known complications. AFCE, a phenotype of acute encephalitis, is a devastating and rapidly progressive neurological disease characterized by an acute onset of alteration in mental status during a febrile phase, leading to fulminant coma, multiorgan failure, and hemophagocytic syndrome. The pathophysiology of influenza-associated cerebral edema is unknown but hypothesized to be caused by cytokine storm [2]. There are few documented cases among the pediatric population but, to the best of our knowledge, this is the first reported case in adults. We present a case of an 18-year-old female with a recent influenza B infection, leading to ARDS and AFCE. 

## Case presentation

We present a case of an 18-year-old female with no significant past medical history who presented to our institution with shortness of breath, fever, and lethargy for one day. She had been discharged from our institution one week earlier after being diagnosed with influenza B and viral myocarditis. Rapid influenza direct antigen testing was positive during the previous admission. Her troponins were elevated at 1.53 ng/mL during that admission, and transthoracic echocardiogram (TTE) and electrocardiogram (EKG) were unremarkable. The patient was hospitalized for two days with no complications, treated with oseltamivir, and later discharged on ibuprofen. Seven days later, she presented with acute respiratory distress, with a respiratory rate of 22 breaths/minute, temperature of 103.0 degrees Fahrenheit, heart rate of 100 beats/minute, and oxygen saturation of 87% on room air. She was also more lethargic on arrival. Initial, pertinent laboratory test results are summarized in Table 1 and Table 2.

**Table 1:** Initial arterial blood gas results PaO2: Partial pressure of oxygen; PaCO2: Partial pressure of carbon dioxide; FiO2: Fraction of inspired oxygen; HCO3: Bicarbonate; O2SAT: Oxygen saturation

Arterial blood gas (reference ranges)	Initial value
pH (7.35-7.45)	7.48
PaO2 (75-100 mmHg)	52
PaCO2 (35-45 mmHg)	25
PaO2/FiO2 ratio (< 300mmHg)	247
HCO3 (22 to 26 mEq/L)	19
O2Sat (95-100%)	87

**Table 2:** Initial laboratory values WBC: White blood cell count; ESR: Erythrocyte sedimentation rate; CRP: C-reactive protein

Test (reference ranges)	Initial value	Follow-up value (if applicable)
Lactic acid (0.5-2.2 mmol/L)	4.8	2.3
Troponin I (<0.028 ng/mL)	0.097	
WBC (4.00 - 12.00 X 10^3^/mcL)	1.2	
ESR (<20 mm/h)	10	20
CRP (<0.50 mg/dL)	0.7	0.4

**Figure 1:**
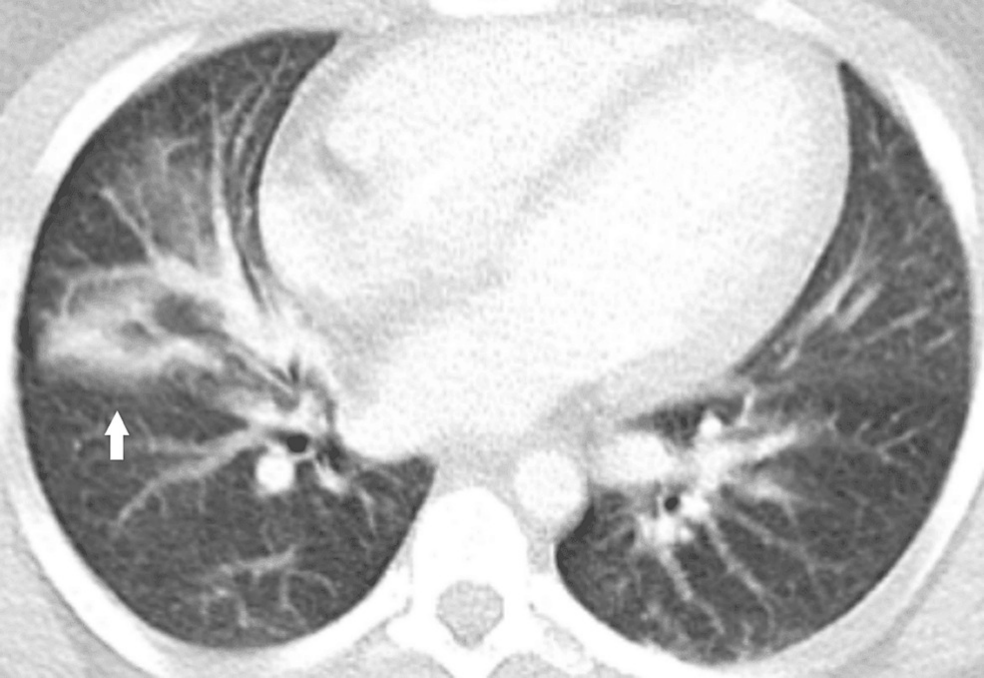
Chest CTA on admission showing a moderate linear consolidation in the right middle lobe (arrow) CTA: computed tomography angiography

On admission to the intensive care unit (ICU), a high-flow nasal cannula was administered at 40 liter/minute with a fraction of inspired oxygen (FiO2) of 40% in combination with budesonide and ipratropium/albuterol inhalers. Empiric antibiotics, including ceftriaxone, cefepime, and vancomycin, were also initiated with intravenous fluids. Chest computed tomography angiography (CTA) revealed a moderate linear consolidation in the right middle lobe and a small linear consolidation in the left lower lobe (Figure 1). Brain computed tomography (CT) scan was unremarkable for any acute abnormality. On day two, lactic acid was found to be trending down at 2.3 mmol/L, but the patient became more lethargic. Acyclovir was also started after suspicion of herpes simplex virus (HSV) encephalitis. The patient was initially awake and alert in the morning but rapidly progressed to a very low full outline of unresponsiveness (FOUR) score later in the day and subsequently was intubated. 

**Figure 2:**
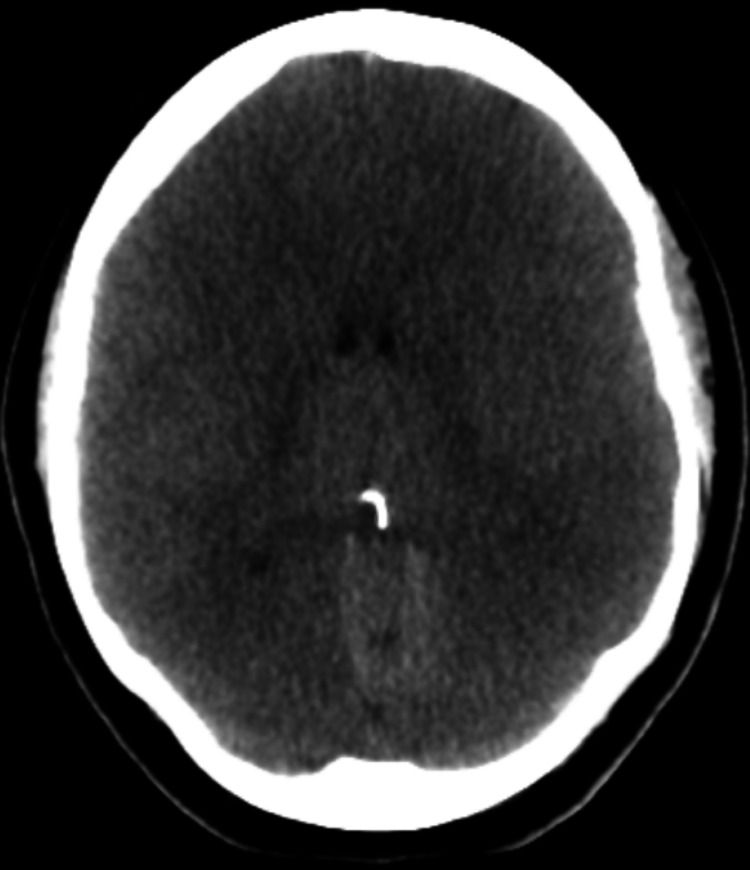
CT of the brain on hospitalization day three showing severe diffuse cerebral edema CT: computed tomography

**Figure 3:**
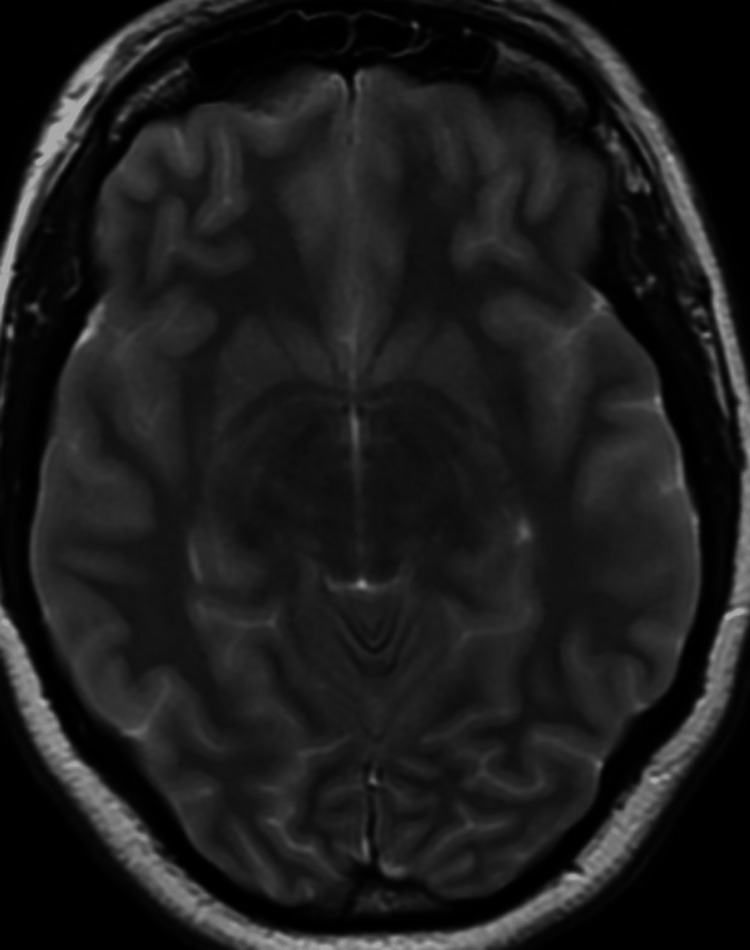
Magnetic resonance fast relaxation fast spin echo T2-weighted images of the brain on day three of admission Magnetic resonance imaging of the brain showing diffuse fast relaxation fast spin echo T2-weighted (FRFSE T2) hyperintensities throughout the cortex, subcortical white matter, deep gray matter with effacement of the cortical sulci, along with diffusion hyperintensity diffusely suggestive of severe adult hypoxic-ischemic injury.

**Figure 4:**
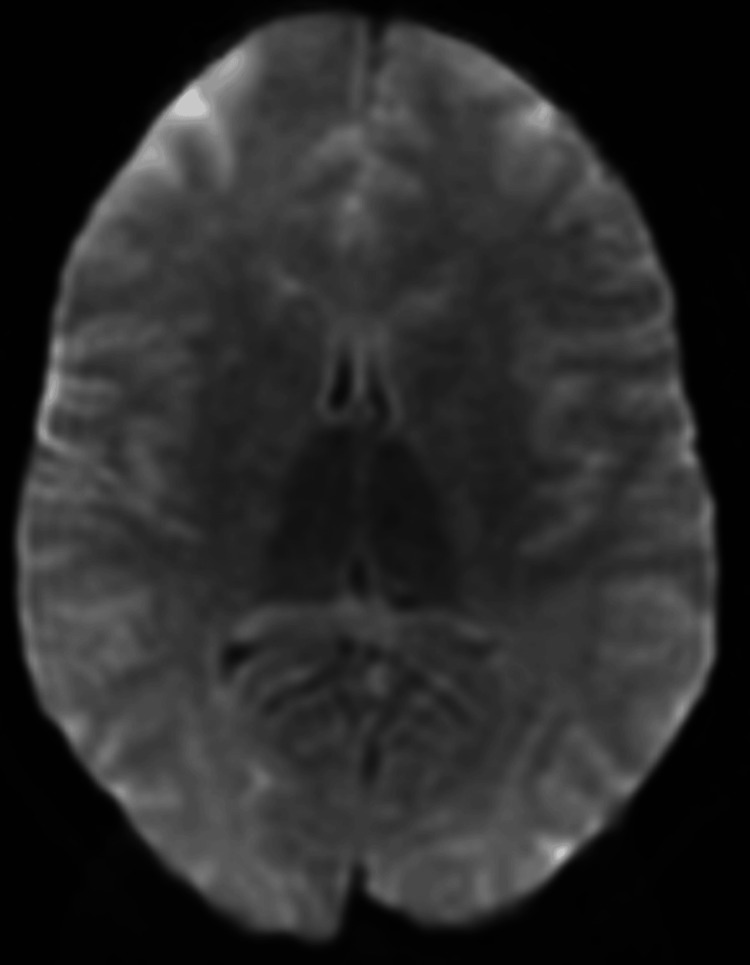
Magnetic resonance diffusion-weighted image on day three of admission showing diffusion hyperintensity diffusely

**Figure 5:**
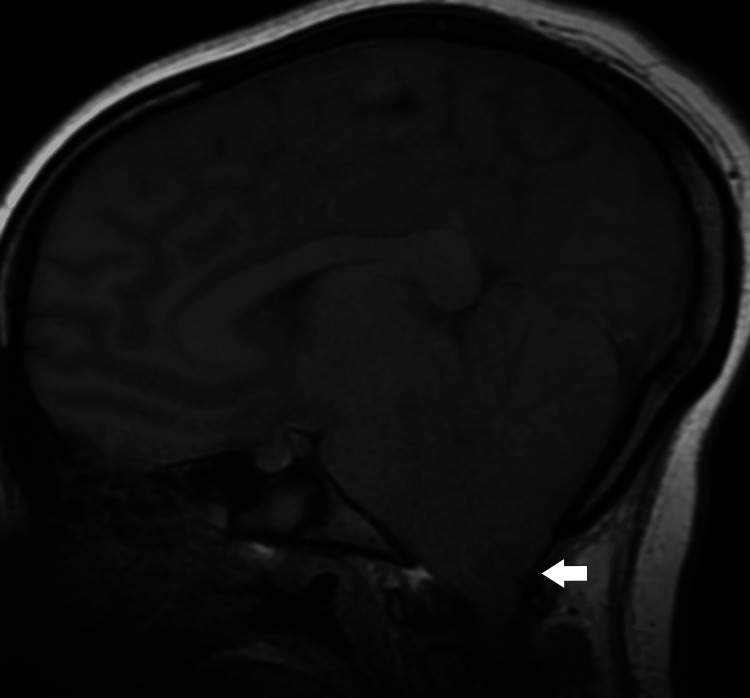
Magnetic resonance sagittal (T1/FLAIR) image on day three of admission showing cerebellar tonsillar herniation in brain (arrow) FLAIR: fluid-attenuated inversion recovery

**Figure 6:**
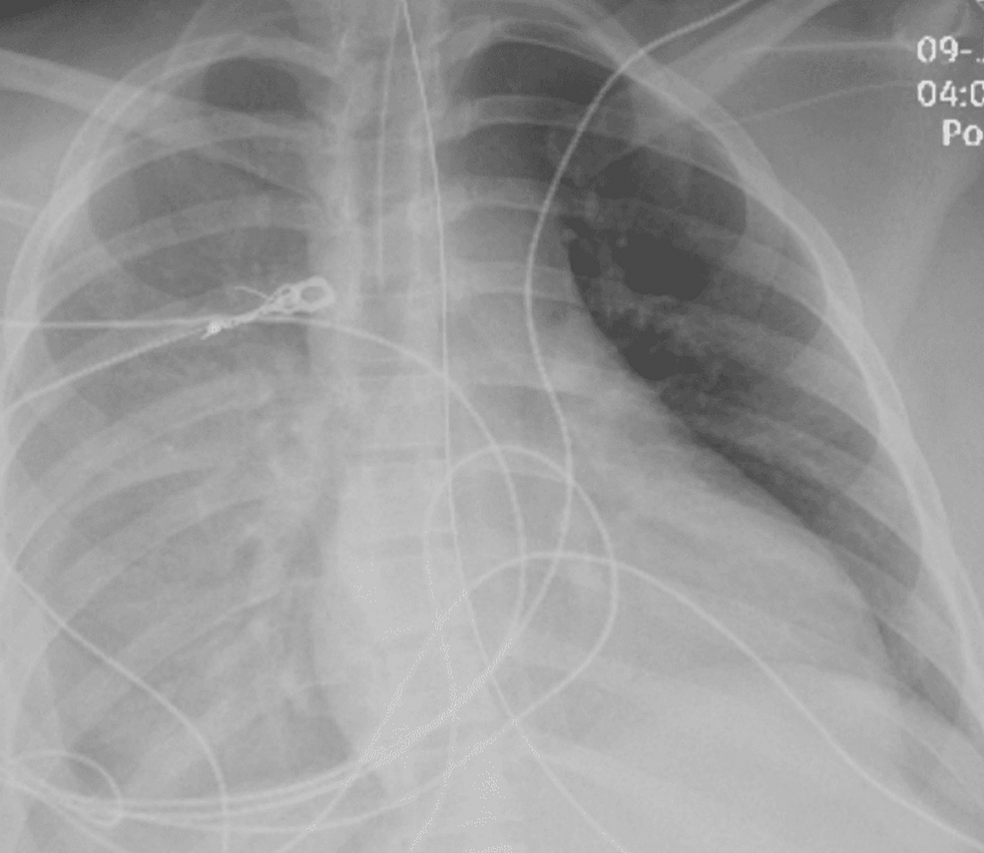
Chest X-ray on day three of admission showing near-complete opacification of the right hemithorax

On day three, after sedation had been stopped, dilated and nonreactive pupils were noted on physical examination. Repeat CT of the brain, completed almost 36 hours after the initial CT of the brain, showed severe diffuse cerebral edema suggestive of anoxic brain injury (Figure 2). Magnetic resonance imaging (MRI) of the brain showed diffuse fast relaxation fast spin echo T2-weighted (FRFSE T2) and diffusion-weighted (DWI) images with hyperintensities throughout the cortex, subcortical white matter, deep gray matter with effacement of the cortical sulci, cerebellar tonsillar herniation along with diffusion hyperintensity diffusely suggestive of severe adult hypoxic-ischemic injury (Figure 3, Figure 4). Cerebellar tonsillar herniation was noted on T1/FLAIR sagittal images of the brain (Figure 5). Chest X-ray was ordered to evaluate respiratory status, which revealed near-complete opacification of the right hemithorax (Figure 6). The sodium level increased to 171 mmol/L, which was believed to be due to central diabetes insipidus; thus, dextrose 5% in water (D5W) was started. Brain stem reflexes on subsequent clinical neurological examination were absent. After official confirmation of brain death, further treatment options were considered futile, given the prognosis.

## Discussion

Influenza B leads to outbreaks and epidemics worldwide. Most infections are asymptomatic or mild, but some can cause substantial morbidity and mortality. One of the complications that carries significant mortality is AFCE. AFCE appears to be uncommon, with tertiary institutions likely seeing only one or two cases every few years [3]. We present a case of an 18-year-old female with a recent influenza B infection, leading to acute fulminant cerebral edema and eventually the patient's demise. 

It is believed that cerebral edema progresses through stages, starting with cytotoxic edema, possibly leading to ionic and vasogenic phases. The improper transportation of ions, which eventually causes water to move into the interstitial compartment, is crucial to the formation of edema [4]. Other diseases or conditions that may result in cerebral edema include neoplastic (primary or metastatic tumor), vascular (hemorrhagic and ischemic stroke), infectious (cerebral abscess, meningitis), toxic (lead/heavy metal encephalopathy), hypoxic-ischemic, and metabolic (hypo-osmolarity, Reye syndrome) processes. In our case, none of these disease processes were present. 

Based on previous case reports and cohorts, it is believed that AFCE is a complex condition that involves various factors. It is thought that both cytotoxic edema (caused by hypoxic-ischemic injury) and vasogenic edema (which may be linked to a systemic inflammatory state) contribute to the development of the condition. These factors may affect the permeability of the blood-brain barrier, which can result in significant cerebral edema and herniation [3]. One cohort study of children with AFCE following severe acute respiratory syndrome coronavirus 2 (SARS-CoV-2) observed that all patients had shock initially, and the inflammatory biomarkers were significantly elevated [2]. An autopsy of an eight-year-old girl with coronavirus disease 2019 (COVID-19) and fulminant cerebral edema demonstrated global brain edema and no evidence of encephalitis, vasculitis, vascular thrombosis, or perivascular demyelination, suggesting a para-infectious mechanism of edema [5]. In these studies, the absence of SARS-CoV-2 RNA in the cerebrospinal fluid (CSF) and brain parenchyma suggested no direct viral invasion of the brain [5,6]. Mutations in carnitine palmitoyl transferase (CPT) II enzyme leading to cerebral edema have been reported in Asian children (specifically from Japan and China) with influenza [7-9]. The CPT enzyme transports long-chain fatty acids from the cytosolic compartment to the mitochondrial matrix to undergo β-oxidation. With these mutations, mitochondrial adenosine triphosphate (ATP) utilization failure occurs during high fever, potentially leading to cerebral edema.

In a case series of four children, with an age range of two to seven years, who presented with altered mental status, vomiting, and neuroimaging suggestive of cerebral edema, one of the patients was positive for Influenza, two had acute hemorrhagic leukoencephalitis, the other two had no identifiable cause. Two patients had normal initial brain imaging, but repeat imaging within 15 hours showed cerebral edema. Only one of them survived [10].

In our patient, inflammatory markers such as erythrocyte sedimentation rate (ESR) and C-reactive protein (CRP) were within normal ranges throughout the hospitalization, suggesting that systemic inflammation was absent. Hypoxic-ischemic injury is unlikely as desaturation below 87% recorded on admission, which was addressed and resolved immediately, was not present during hospitalization. Furthermore, adequate perfusion pressures were maintained throughout hospitalization. Occasional hypotensive episodes were noted; the lowest blood pressure recorded was 87/50 mmHg on day three, which was also addressed and resolved after two hours. On day one and day three, our patient had a peak temperature of 103.0 degrees Fahrenheit, thus supporting the hypothesis that cerebral edema may be caused by mitochondrial ATP utilization failure during times of high fever. The direct viral invasion of the central nervous system could not be established in our case, as CSF samples were never obtained.

## Conclusions

The pathophysiology of AFCE remains complex and not entirely understood. It is likely that a combination of factors, including hypoxic-ischemic injury, systemic inflammatory response, and potential genetic predisposition, might contribute to the development and progression of this severe neurologic complication. To our knowledge, this is the first reported case of this complication caused by influenza B in the adult population. It highlights the need for continued research in children and adults to understand the underlying pathogenesis of AFCE, identify potential risk factors, and explore novel therapeutic interventions.
